# Laboratory diagnosis and management of COVID-19 cases: creating a safe testing environment

**DOI:** 10.1186/s12879-021-06806-0

**Published:** 2021-10-29

**Authors:** Titilayo Tosin Lekan-Agunbiade, Olalekan Isaiah Agunbiade

**Affiliations:** Midland Regional Hospital, Portlaoise, Ireland

**Keywords:** Medical scientist, Laboratory-testing, Laboratory safety, COVID-19 diagnosis, Awareness, Safety enabling factors, Personal efforts, Laboratory environment

## Abstract

**Background:**

COVID-19 disease has had a profound impact worldwide since it was discovered in Wuhan, China, in December 2019. Laboratory testing is crucial to prompt identification of positive cases, initiation of treatment and management strategies. However, medical scientists are vulnerable to infection due to the risk of exposure in the laboratory and the community. This study sought to determine the awareness of laboratory safety measures, assess the personal efforts of medical scientists in creating a safe laboratory environment for testing and examine the laboratory safety enabling factors.

**Methods:**

The data used for the study were generated among medical scientists in Nigeria through an internet-broadcasted questionnaire and were analyzed using IBM *SPSS* Statistics (version 25).

**Results:**

The majority of the respondents had a high awareness of laboratory safety measures (60.3%) and demonstrated good personal efforts in creating a safe laboratory testing environment (63%). The level of awareness of laboratory safety measures was significantly associated with respondents’ level of education (χ^2^ = 6.143; p = 0.046) and influences respondents’ efforts in creating a safe laboratory testing environment (p = 0.007). However, just a few respondents could convincingly attest to the availability of adequate and appropriate PPE with proper utilization training (45.1%), adequate rest and other welfare packages (45.8%) as well as access to appropriate Biological Safety Cabinets (BSCs) and other essential equipment in their laboratories (48.8%). Furthermore, a significant association existed between the availability of laboratory safety enabling factors and respondents’ efforts in creating a safe environment for testing with the p-value ranging between < 0.0001 and 0.003.

**Conclusion:**

This study revealed that despite the high awareness of safety measures and good personal efforts of the study participants in creating a safe laboratory-testing environment, there was poor availability of safety facilities, equipment, support and welfare packages required to enhance their safety. It is, therefore, crucial to provide necessary laboratory biosafety equipment and PPE in order not to compromise medical scientists’ safety as they perform their duties in COVID-19 pandemic response.

**Supplementary Information:**

The online version contains supplementary material available at 10.1186/s12879-021-06806-0.

## Background

Since the turn of the third decade in the new millennium, the COVID-19 pandemic has been ravaging the world, after it was first discovered in Wuhan. To date, many lives have been lost to this deadly virus—as of 10 January 2020, there are more than 90 million confirmed cases globally, and more than 1.9 million deaths [1]. In Nigeria, there are 99,063 confirmed cases and 1,350 deaths [2].

The governments and health institutions of various countries have put many strategic plans in place to aid their responses to contain and manage the effect of the pandemic on the health of their populace. Central and pertinent to these responses are the roles that the medical scientists play as part of the multidisciplinary healthcare team in ensuring reliable, accurate and timely diagnosis, monitoring of positive patients, therapeutic drug monitoring/surveillance, confirmation of recovery, validation of testing protocols, invention and development of novel vaccines. Reports showed that most of the COVID-19 patients require regular laboratory testing to ensure adequate staging, prognosis, therapeutic monitoring and epidemiological surveillance [3].

### COVID-19 testing and management recommendations by international federation of clinical chemistry and laboratory medicine (IFCC)

IFCC recommendations were given to support laboratory practices with a particular focus on clinical laboratories in developing countries as the fight against COVID-19 continues. IFCC advised to carefully select appropriate testing method bearing in mind that the performance of some point–of–care assays has not been well demonstrated and a minimum of 2 gene targets were recommended for molecular assays to minimize false-negative results. It was stated that sample types and sample collection must follow the manufactures’ recommendations, as well as the verification of analytical and clinical performance of regulatory-approved molecular test must be done before routine use. Participation in relevant Quality Assurance Programs was also recommended as essential [4].

### World health organization’s (WHO) recommendations on laboratory safety standards

WHO recommends that every laboratory conducting COVID-19 testing should be appropriately equipped, staff must be properly trained in the technical and safety procedures as well as in the essential containment practices. Risk assessment must be conducted to ascertain the laboratory’s competency to perform testing safely, to identify and mitigate risk, as well as to put necessary and appropriate risk control measures in place. WHO also emphasizes that good microbiological practices and procedures (GMPP) should be adequately observed and that the preliminary processing of all samples should be performed in a biological safety cabinet (BSC) validated for use and properly maintained while routine viral testing of specimens should be performed in a biosafety Level 2 (BSL-2) laboratory with a certified Class II BSC. Procedures that involve viral concentration are expected to be done in a BSL-2 laboratory with unidirectional airflow and BSL-3 precautions. Biosafety Level 3 (BSL-3) laboratory is required for virus isolation in cell culture and disinfectants with known potency against enveloped viruses such as hypochlorite or phenolic compounds are required to be utilized in the laboratory to reduce contamination. Procedures that minimize aerosols and droplets generation must be adopted and appropriate personal protective equipment (PPE) must be worn by all laboratory staff handling specimens. Specimens from suspected or confirmed COVID-19 patients must be placed in a secondary container to reduce spillage or breakage and must be transported as Category B, UN3373 “Biological Substance” while viral cultures or isolates are required to be transported as Category A, UN2814, “infectious substance, affecting humans” [5].

A survey conducted by the International Federation of Clinical Chemistry and Laboratory Medicine (IFCC) involving participants from 86 worldwide regions and countries reveals some additional safety measures observed by some laboratories which include, test menu restrictions, temperature monitoring of staff, increasing disinfection frequency, and splitting of staff teams [6].

Due to the risk of exposure in the laboratory in addition to the risk of exposure in the community, the laboratory staff is vulnerable to infection. Reports indicate that more than 90,000 healthcare workers are infected worldwide less than 6 months into the pandemic [7] and the numbers are yet increasing. Therefore, the importance of ensuring a safe environment in the laboratory cannot be overemphasized.

This study was designed to determine the awareness of laboratory safety measures among medical scientists, assess the personal efforts of medical scientists in creating a safe testing environment, and to examine the laboratory safety-enabling factors.

## Methods

This descriptive cross-sectional survey involving 131 medical scientists from Nigeria was conducted from May 14 to May 28, 2020. The recruitment process was designed to capture participants from the six- geopolitical zones in Nigeria using the Snowball sampling method. To be eligible for recruitment, participants must be medical scientists practicing in Nigeria and must give written informed consent to participate in the survey.

### Survey instrument

Internet-broadcasted structured questionnaire was developed for the survey. It was designed using Google forms and the link was sent to participants through WhatsApp. The prospective participants were encouraged to send out the link to other scientists and online platforms. The questionnaire was pretested by sending the unpublished online questionnaires to a cohort of 19 medical scientists in Nigeria. Questions that pre-test participants found unclear, difficult to answer, irrelevant, or out of scope of the study were noticed, revised, and re-tested. Internal consistency of 0.8 was achieved for the instrument using the Cronbach’s Alpha method. All responses were captured on the six-point Likert scale, however strongly disagree and disagree responses as well as strongly agree and agree responses were merged as disagree and agree, respectively in the report.

The 4 sections of the questionnaire used explored respondents’ demographics (contained six items), level of awareness of laboratory safety measures and the personal efforts in creating a safe testing environment (contained 10 items each), as well as laboratory safety enabling factors (contained 12 items).

### Data analysis

The study participants’ responses were coded from 1 to 6 based on 6-points Likert Scale (Starting from strongly disagree to strongly agree). The level of awareness of laboratory safety measures and personal efforts in creating a safe laboratory environment had a maximum score of 60 while overall mean scores were 49.7 (± 4.8) and 48.5 (± 5.9), respectively. These mean scores were used as the benchmark to categorize the level of awareness and personal efforts as either good or poor.

The data analysis explored frequency distribution, mean ± SD, and graphs (Descriptive statistics), bivariate analysis (Chi-square (χ^2^) test, and multivariate linear regression model (inferential statistics). The analysis was done using IBM *SPSS Statistics* (version 25) with significance and confidence levels set at p < 0.05 and 95%, respectively.

## Results

One hundred and one participants were involved in this study. Eligible participants were medical scientists working in Nigeria. Respondents’ socio-demographic characteristics, awareness of laboratory safety measures, personal efforts in creating a safe laboratory testing environment, association between awareness of laboratory safety measures and demographics as well as the laboratory safety enabling factors were reported. In addition, the influence of awareness and years of experience on personal efforts of respondents to create a safe testing environment as well as the association between personal efforts to create a safe laboratory testing environment and laboratory safety enabling factors were reported.

### Socio-demographic characteristics of subjects

The modal age group of the respondents was between 20 and 29 years old (42.0%) while the mean age of the respondents was 32.5 ± 9.9 years. The majority of the respondents were males (64.1%), graduates (64.1%), Christians (81.7%), with years of work experience between 1 and 9 years (70.2%) and with the present level at work between levels 1 and 5 (61.1%) (Table1).

**Table 1:** Socio-demographic characteristics of Respondents; n = Total Number of Respondents

	Frequency (n = 131)	Percentage (100%)
Age (in grouped years): [32.5 ± 9.9 years]		
< 20 years	2	1.5
20–29 years	55	42.0
30–39 years	44	33.6
40–49 years	20	15.3
50–59 years	10	7.6
Gender		
Male	84	64.1
Female	47	35.9
Religion		
Christianity	107	81.7
Islam	23	17.6
Agnostic	1	0.8
Highest education		
Graduate	84	64.1
Post-graduate	39	29.8
Fellowship	8	6.1
Years of experience (grouped): [7.5 ± 8.1 years]		
< 1 year	7	5.3
1–9 years	92	70.2
10–19 years	16	12.2
20–29 years	12	9.2
30–39 years	4	3.1
Present post/Level at work		
1–5	80	61.1
6–10	21	16.0
11–15	16	12.2
> 15	14	10.7
Total	131	100.0

### Awareness of laboratory safety measures

Most of the respondents strongly agreed that laboratory safety is important and essential to the success of any medical laboratory (98.5%) and access to appropriate Biological Safety Cabinets (BSCs) is critical in the containment of SARS-CoV-2 (87%) (Table 2). The overall mean awareness score was 49.7 (± 4.8) and the majority of respondents (60.3%) scored above the mean; therefore, they were classified as having a high awareness of laboratory safety measures (See Additional file 4: Fig. S1). The awareness level of laboratory safety measures was significantly associated with the level of education (χ^2^ = 6.143; p = 0.046) of medical scientists (See Additional file 1: Table S1). The awareness level of laboratory safety measures also had a significant influence on the personal efforts of the study participants in creating a safe laboratory testing environment (p = 0.007). (See Additional file 2: Table S2).

**Table 2:** Awareness of laboratory safety measures

Awareness variables	Strongly agree n (%)	Agree n (%)	Slightly agree n (%)	Slightly disagree n (%)	Disagree n (%)	Strongly disagree n (%)
Laboratory safety is an important and essential component to the success of any medical laboratory	118 (90.1)	11 (8.4)	2 (1.5)	0 (0.0)	0 (0.0)	0 (0.0)
It is important to understand and follow instructions in the Laboratory Safety Manual	118 (90.1)	13 (9.9)	0 (0.0)	0 (0.0)	0 (0.0)	0 (0.0)
Laboratory safety practices are never impacted by biological, chemical, radiological, fire, and electrical hazards	8 (6.1)	15 (11.5)	4 (3.1)	4 (3.1)	47 (35.9)	53 (40.5)
It is critical to have access to SOPs that document safety procedures	86 (65.6)	25 (19.1)	6 (4.6)	0 (0.0)	9 (6.9)	5 (3.8)
Processes that emit vapors, gasses, or fumes should be adequately captured by local ventilation (hoods, snorkel)	80 (61.1)	38 (29.0)	7 (5.3)	1 (0.8)	3 (2.3)	2 (1.5)
It is important for laboratory equipment with potential hazards routinely inspected and maintained or serviced as recommended	106 (80.9)	24 (18.3)	1 (0.8)	0 (0.0)	0 (0.0)	0 (0.0)
It is important to have access to chemical/biological spill kits	91 (69.5)	32 (24.4)	2 (1.5)	1 (0.8)	3 (2.3)	2 (1.5)
It is important to have access to fully stocked first-aid kits	113 (86.3)	14 (10.7)	1 (0.8)	0 (0.0)	2 (1.5)	1 (0.8)
Good housekeeping practices are less essential in ensuring laboratory safety	16 (12.2)	14 (10.7)	8 (6.1)	8 (6.1)	46 (35.1)	39 (29.8)
Access to appropriate Biological Safety Cabinets (BSCs) is critical in the containment of SARS-CoV-2	87 (66.4)	27 (20.6)	12 (9.2)	1 (0.8)	1 (0.8)	3 (2.3)

### Personal efforts in creating a safe laboratory-testing environment

Table 3 showed that most of the respondents had participated in laboratory safety (75.6%) and PPE training (64.2%) in the last 1 year. The majority (77.1%) of the study participants knew the emergency contact numbers to use during emergencies, as well as how and when to report accidents, incidents or near misses in the laboratory (91.6%). The results also demonstrated that almost all of the respondents always use personal protective equipment (PPE) (90.1%), regularly carry out adequate decontamination of bench surfaces (90.8%), clearly label all containers with their contents (97%) and often wash hands (97%) when working in the laboratory. Not less than 93.9% of respondents attested to always removing PPE before leaving the laboratory and refrained from eating, drinking, applying cosmetics and handling contact lenses in the laboratory. The overall mean personal effort score was 48.5 (± 5.9) (Additional file 5: Fig. S2) and most of the survey participants (63%) had good personal safety practices as they scored above the mean.

**Table 3:** Personal efforts in creating a safe laboratory-testing environment

	Strongly agree n (%)	Agree n (%)	Slightly agree n (%)	Slightly disagree n (%)	Disagree n (%)	Strongly disagree n (%)
In the last 1 year, I have participated in laboratory safety training	50 (38.2)	49 (37.4)	13 (9.9)	5 (3.8)	8 (6.1)	6 (4.6)
I know the emergency contact numbers to contact when an emergency occurs	53 (40.5)	48 (36.6)	14 (10.7)	0 (0.0)	11 (8.4)	5 (3.8)
I know how and when to report accidents, incidents or near-misses in the Laboratory	57 (43.5)	63 (48.1)	7 (5.3)	1 (0.8)	1 (0.8)	2 (1.5)
I clearly label all containers with their contents	96 (73.3)	31 (23.7)	2 (1.5)	1 (0.8)	1 (0.8)	0 (0.0)
I have completed PPE training within the last 1 year	50 (38.2)	34 (26.0)	17 (13.0)	6 (4.6)	17 (13.0)	7 (5.3)
I always use personal protective equipment (PPE) when working in the laboratory	80 (61.1)	38 (29.0)	9 (6.9)	0 (0.0)	4 (3.1)	0 (0.0)
I regularly carry out adequate decontamination of bench surfaces, all wastes and other materials in the laboratory	86 (65.6)	33 (25.2)	7 (5.3)	3 (2.3)	2 (1.5)	0 (0.0)
I eat, drink, apply cosmetics, and handle contact lenses in the laboratory	1 (0.8)	3 (2.3)	1 (0.8)	3 (2.3)	29 (22.1)	94 (71.8)
I wash my hands often – especially after handling infectious materials, before leaving the laboratory working areas, and before eating	113 (86.3)	14 (10.7)	1 (0.8)	0 (0.0)	3 (2.3)	0 (0.0)
I always remove my PPE before leaving the laboratory	103 (78.6)	20 (15.3)	4 (3.1)	1 (0.8)	3 (2.3)	0 (0.0)

### Laboratory safety enabling factors

This study revealed that most respondents opined that safety enabling factors such as sufficiently trained medical scientists (58%), reviewed, updated protocols and working practice policies (74%), training and awareness plans, as well as Standard Operating Procedure (SOP) (74.1%) were available in their laboratories. The majority also confirmed that staff was informed of the risk associated with SARS-CoV-2 infection (80.9%), that the process for incident reporting and investigation existed (72.5%) and that sufficient space was available in the laboratories where they work (69.5%). More than half of the respondents agreed that adequate supplies of required disinfectants and other materials were ensured (67.2%), that procedures were in place to ensure materials can be transported safely to and from the laboratory (67.2%) and that good general security controls were in place including those required to address out of hours work times (63.3%).

However, just a few respondents could convincingly attest to the availability of adequate and appropriate PPE with proper utilization training (45.1%), adequate rest and other welfare packages (45.8%), as well as access to appropriate Biological Safety Cabinets (BSCs) and other essential equipment in their workplaces (48.8%). (Table 4).

**Table 4:** Laboratory safety enabling factors

	Strongly agree n (%)	Agree n (%)	Slightly agree n (%)	Slightly disagree n (%)	Disagree n (%)	Strongly disagree n (%)
Sufficiently trained Medical scientists are available in my place of work	43 (32.8)	33 (25.2)	23 (17.6)	7 (5.3)	15 (11.5)	10 (7.6)
Reviewed, updated protocols and working practice policies are available and communicated (e.g. a safe work practices, decontamination) in my place of work	49 (37.4)	48 (36.6)	21 (16.0)	4 (3.1)	8 (6.1)	1 (0.8)
Training and awareness plans, as well as Standard Operating Procedure (SOP) compliance programs are in place for all staff	55 (42.0)	42 (32.1)	16 (12.2)	8 (6.1)	8 (6.1)	2 (1.5)
Adequate and appropriate PPEs are supplied (including disposable gloves, solid-front or wrap-around gowns, or coveralls with sleeves that fully cover the forearms, eye protection (goggles or face shield), and respiratory protection (US6NIOSH-certified N95 or equivalent, or higher protection), are available and staff are trained in their use	36 (27.5)	23 (17.6)	31 (23.7)	7 (5.3)	15 (11.5)	19 (14.5)
Provisions for adequate rest and other welfare issues (e.g. workplace stress, concern for family members) are available in my place of work	32 (24.4)	28 (21.4)	23 (17.6)	14 (10.7)	18 (13.7)	16 (12.2)
All staff (i.e. scientific and support) are informed of the risk associated with SARS-CoV-2 infection, symptoms, reporting procedures and support from the organization/hospital in the event of illness	65 (49.6)	41 (31.3)	14 (10.7)	5 (3.8)	4 (3.1)	2 (1.5)
Process for incident reporting and investigation exists in my place of work	52 (39.7)	43 (32.8)	22 (16.8)	8 (6.1)	6 (4.6)	0 (0.0)
Sufficient space, including storage of specimens and other materials (e.g. waste), is available in my place of work	50 (38.2)	41 (31.3)	23 (17.6)	4 (3.1)	9 (6.9)	4 (3.1)
Access to appropriate Biological Safety Cabinets (BSCs) and other essential equipment is ensured in my place of work	37 (28.2)	27 (20.6)	31 (23.7)	11 (8.4)	18 (13.7)	7 (5.3)
Adequate supplies of required disinfectants and other materials are ensured at my place of work	45 (34.4)	43 (32.8)	24 (18.3)	4 (3.1)	13 (9.9)	2 (1.5)
Procedures are in place to ensure materials can be transported safely to and from the laboratory	47 (35.9)	45 (34.4)	26 (19.8)	6 (4.6)	6 (4.6)	1 (0.8)
Good general security controls are in place including those required to address out of hours work times	37 (28.2)	46 (35.1)	25 (19.1)	10 (7.6)	6 (4.6)	7 (5.3)

Interestingly, a strong association existed between respondents’ personal effort in creating a safe laboratory testing environment and all the laboratory safety-enabling factors explored in this study (See Additional file 3: Table S3).

## Discussion

Medical scientists are constantly at risk of exposure to infectious agents in the course of their work especially in the era of the COVID-19 pandemic where laboratory test results underscore efficient pandemic response. Laboratory safety requires an awareness of exposure risks, compliance to safe laboratory practices, adherence to standard operating procedures and use of containment equipment in the laboratory. Studies have shown that when laboratory staff are aware and adhere to the recommended safety precautions, the risk for laboratory-acquired infections becomes lower [8].

However, awareness and biosafety are big issues in laboratory settings in developing countries as standard operating procedures (SOPs) are lacking and less efficient [9]**.**

With Nigeria coping with the challenges of the COVID-19 pandemic, ensuring a safe testing environment is the core of laboratory diagnosis and management of COVID-19 cases.

This study was designed to determine the awareness of laboratory safety measures, assess personal efforts of medical scientists in creating a safe environment for testing, and examine the laboratory safety-enabling factors.

Most of the study participants (60.3%) were found to possess high-level of awareness of laboratory safety measures and the level of awareness of laboratory safety measures was significantly associated with their level of education (χ^2^ = 6.143; p = 0.046). Respondents displayed in-depth awareness of the importance of laboratory safety practice; understanding and following instructions in the laboratory safety manual as well as standard operating procedures; local ventilation; proper maintenance of laboratory equipment, access to chemical/biological spill kits and fully stocked first-aid kits.

In addition, study participants had a good orientation about good housekeeping practices and the importance of access to appropriate Biological Safety Cabinets (BSCs) in the containment of SARS-CoV-2. These findings are incongruent with the results of the study conducted among laboratory staff of two public health facilities in Nigeria. According to this study, many respondents (41.5%) were found to be unaware of laboratory safety practices and 25.4% of respondents do not observe safety practice in the laboratory [10]^.^ The difference in the level of awareness in these two studies may be due to the increased laboratory safety education, training and campaigns especially in this era of the COVID-19 pandemic.

The study found that the majority of the respondents (63%) showed good personal effort in creating a safe laboratory testing environment by regularly participating in laboratory safety and PPE training, by prompt reporting of accidents, incidents or near-misses in the laboratory and adequate decontamination of bench surfaces, all generated wastes and other materials in the laboratory. Most of the respondents also carried out proper labeling of containers with their contents and regular hand washing. Most respondents attested to regular use of personal protective equipment (PPE) when working in the laboratory and not eating, drinking, applying cosmetics or handling contact lenses in the laboratory. These personal efforts from medical scientists reinforce a safe environment in the laboratory, which enhances the provision of quality, efficient and effective laboratory services which are essential in productive COVID-19 pandemic response. The study conducted among personnel who worked in various laboratories and hospitals in Denizli, Turkey yielded similar results where study participants displayed good personal efforts in enhancing optimum laboratory safety [11].

It was also revealed in this study, that safety awareness has a positive impact on safety practice as awareness of laboratory safety measures had a significant influence on personal efforts of medical scientists in creating a safe laboratory-testing environment. This is in agreement with the result of a study in Pakistan in which the participating healthcare workers showed good knowledge/awareness of COVID-19 disease and displayed good safety practices [12].

Even though most of the medical scientists in this study demonstrated good personal efforts in creating a safe laboratory-testing environment, there were, however, inadequate supply of appropriate PPEs as well as poor training in their use, inadequate provisions for rest and other welfare packages, poor access to appropriate Biological Safety Cabinets (BSCs) and other essential equipment. This discovery is an aberration to the recommended WHO laboratory biosafety standard and may jeopardize the safety of medical scientists, reduce the chance of obtaining timely, accurate and reliable laboratory results which are important for effective and efficient COVID-19 pandemic responses such as diagnosing, managing, determining treatment and prognostic outcomes, as well as the overall patient safety. This is consistent with previous findings in some research facilities in Nigeria in which study participants attested to poor availability of PPE and no access to biosafety level (BSL)-1–4 facilities [13]. This result also agrees with the report of the survey conducted by the International Federation of Clinical Chemistry and Laboratory Medicine IFCC in which the majority of the laboratory staff work without wearing face masks, eye goggles, disposable gowns or use appropriate safety cabinets even when performing aerosol-generating procedures [14, 15].

Interestingly, a strong association existed between the personal efforts of respondents in creating a safe laboratory testing environment and laboratory safety enabling factors. This further substantiates the importance of availability and accessibility of safety equipment, tools, supplies and materials in the laboratory.

## Conclusion

The respondents displayed a high level of awareness of safety standards and great personal efforts in creating a safe laboratory-testing environment by observing safety protocols. However, it was evident that there was poor availability of safety facilities, equipment, support and welfare packages required to enhance the safety of these medical scientists as they play their roles as part of front line workers in the battle against the deadly COVID-19 disease. The safety of medical scientists should constitute a major concern as the pressure to increase COVID-19 testing with short turnaround times increases. In order not to compromise the safety of medical scientists and the efficiency of laboratory test results, we advocate for the provision of biosafety devices, PPEs in medical laboratories across Nigeria and zeal boosting welfare and support packages especially in this critical time in the world's history. Adequate training in the use of PPEs, in laboratory biosafety practices and maintenance of biosafety equipment, should be prioritized.

Lastly, policies on safety practices should be reinforced and enforced.

However, it is important to note that the participants’ recruitment method used relies majorly on personal and social relationships to reach potential participants instead of a more systematic sampling method. It also has the potential for sample clustering which may make generalization of the study findings difficult.

In addition, there is increased likelihood of recall bias as participants’ responses were largely dependent on the honest report of the real-life experiences. Therefore, more studies with systematic sampling methods, multi-dimensional measures and expanded inclusion such as focus groups, in-depth interviews with the inclusion of medical scientists who are located in rural areas with limited internet access, are warranted.

## Supplementary Information

**Additional file 1: Table S1. **Awareness level of laboratory safety measures in relation to their demographics.

**Additional file 2: Table S2. **Influence of awareness on personal efforts in creating a safe laboratory-testing environment and years of experience.

**Additional file 3: Table S3. **Association between personal effort of medical scientist in creating a safe laboratory-testing environment and laboratory safety enabling factors**.**

**Additional file 4: Figure S1.** Overall level of awareness of laboratory safety measures.

**Additional file 5: Figure S2.** Overall level of personal efforts in creating a safe laboratory testing environment.

## Data Availability

The datasets used and/or analysed during the current study are available from the corresponding author on reasonable request.

## Abbreviations

BSCs: Biological Safety Cabinets
BSL: Bio-Safety Level
IFCC: International Federation of Clinical Chemistry and Laboratory Medicine
PPE: Personal Protective Equipment
SD: Standard Deviation
SOP: Standard Operating Procedure
WHO: World Health Organization
