# Supplementary material for: Laboratory diagnosis and management of COVID-19 cases: creating a safe testing environment
Source: BMC Infect Dis. 2021 Oct 29;21:1114. doi: 10.1186/s12879-021-06806-0 (PMC8554734; doi:10.1186/s12879-021-06806-0)
Supplement: Supplementary file 1 — Additional file 1: Table S1. Awareness level of laboratory safety measures in relation to their demographics. [file 12879_2021_6806_MOESM1_ESM.pdf]

Table 1: Awareness Level of Laboratory Safety Measures In Relation To Their Demographics

**Demographic data**	**Awareness level**		χ^2
	**Low n (%)**	**High n (%)**	
**Age (in grouped years):**			6.967
**<20 years**	2 (3.8)	0 (0.0)	p = 0.138
**20 – 29 years**	26 (50.0)	29 (36.7)	
**30 – 39 years**	16 (30.8)	28 (35.4)	
**40 – 49 years**	6 (3.8)	14 (17.7)	
**50 – 59 years**	2 (3.8)	8 (10.1)	
**Gender:**			0.250
**Male**	32 (61.5)	52 (65.8)	p = 0.710
**Female**	20 (38.5)	27 (34.2)	
**Religion:**			1.286
**Christianity**	40 (76.9)	67 (84.8)	p = 0.526
**Islam**	12 (23.1)	11 (13.9)	
**Agnostic**	0 (0.0)	1 (1.3)	
**Highest Education:**			6.143
**Graduate**	40 (76.9)	44 (55.7)	**p = 0.046**
**Post graduate**	10 (19.2)	29 (36.7)	
**Fellowship**	2 (3.8)	6 (7.6)	
**Years of experience :**			6.177
**<1 year**	4 (7.7)	3 (3.8)	p = 0.186
**1 – 9 years**	41 (78.8)	51 (64.6)	
**10 – 19 years**	3 (5.8)	13 (16.5)	
**20 – 29 years**	3 (5.8)	9 (11.4)	
**30 – 39 years**	1 (1.9)	3 (3.8)	
**Present post/Level at work:**			
**1 – 5**			2.782
**6 – 10**	36 (69.2)	44 (55.7)	p = 0.426
**11 – 15**	6 (11.5)	15 (19.0)	
**>15**	6 (11.5)	10 (12.7)	
	4 (7.7)	10 (12.7)	
Total	**52 (100.0)**	**79 (100.0)**	
