# Supplementary material for: Laboratory diagnosis and management of COVID-19 cases: creating a safe testing environment
Source: BMC Infect Dis. 2021 Oct 29;21:1114. doi: 10.1186/s12879-021-06806-0 (PMC8554734; doi:10.1186/s12879-021-06806-0)
Supplement: Supplementary file 2 — Additional file 2: Table S2. Influence of awareness on personal efforts in creating a safe laboratory-testing environment and years of experience. [file 12879_2021_6806_MOESM2_ESM.pdf]

Table 2: Influence of Awareness on Personal Efforts in Creating a Safe Laboratory-Testing Environment and Years of Experience.

Source	Dependent Variables	Type I Sum of Squares	df	Mean Square	F	p-value
Awareness of laboratory safety measures	Personal efforts in creating a safe Laboratory-testing Environment	249.865	1	249.865	7.529	**0.007**
	Years of experience as a medical scientist	133.298	1	133.298	2.061	0.154
