# Supplementary material for: Laboratory diagnosis and management of COVID-19 cases: creating a safe testing environment
Source: BMC Infect Dis. 2021 Oct 29;21:1114. doi: 10.1186/s12879-021-06806-0 (PMC8554734; doi:10.1186/s12879-021-06806-0)
Supplement: Supplementary file 3 — Additional file 3: Table S3. Association between personal effort of medical scientist in creating a safe laboratory-testing environment and laboratory safety enabling factors. [file 12879_2021_6806_MOESM3_ESM.pdf]

Table 3: Association between Personal Effort of Medical Scientist in Creating a Safe Laboratory-Testing Environment and Laboratory Safety Enabling Factors.

	Correct answers		χ^2 p-value
	Poor Personal efforts n (%)	Good Personal efforts n (%)	
1. Sufficiently trained Medical scientists are available in my place of work	6 (14.0)	37 (86.0)	**<0.0001**
2. Reviewed, updated protocols and working practice policies are available and communicated (e.g. a safe work practices, decontamination) in my place of work	6 (12.2)	43 (87.8)	**<0.0001**
3. Training and awareness plans, as well as Standard Operating Procedure (SOP) compliance programmes are in place for all staff	8 (14.5)	47 (85.5)	**<0.0001**
4. Adequate and appropriate PPEs are supplied (including disposable gloves, solid-front or wrap-around gowns, or coveralls with sleeves that fully cover the forearms, eye protection (goggles or face shield), and respiratory protection (US6NIOSH-certified N95 or equivalent, or higher protection), are available and staff are trained in their use	4 (11.1)	32 (88.9)	**<0.0001**
5. Provisions for adequate rest and other welfare issues (e.g. workplace stress, concern for family members) are available in my place of work	4 (12.5)	28 (87.5)	**<0.0001**
6. All staff (i.e. scientific and support) are informed of the risk associated with SARS-CoV-2 infection, symptoms, reporting procedures and support from the organization/hospital in the event of illness	13 (20.0)	52 (80.0)	**<0.0001**
7. Process for incident reporting and investigation exists in my place of work	6 (11.5)	46 (88.5)	**<0.0001**
8. Sufficient space, including storage of specimens and other materials (e.g. waste) is available in my place of work	6 (12.0)	44 (88.0)	**<0.0001**
9. Access to appropriate Biological Safety Cabinets (BSCs) and other essential equipment is ensured in my place of work	2 (5.4)	35 (94.6)	**<0.0001**
10. Adequate supplies of required disinfectants and other materials are ensured at my place of work	7 (15.6)	38 (84.4)	**<0.0001**
11. Procedures are in place to ensure materials can be transported a safely to and from the laboratory	4 (8.5)	43 (91.5)	**<0.0001**
12. Good general security controls are in place including those required to address out of hours work times	3 (8.1)	34 (91.9)	**<0.0001**
