# Supplementary figures and images for: Laboratory diagnosis and management of COVID-19 cases: creating a safe testing environment
Source: BMC Infect Dis. 2021 Oct 29;21:1114. doi: 10.1186/s12879-021-06806-0 (PMC8554734; doi:10.1186/s12879-021-06806-0)

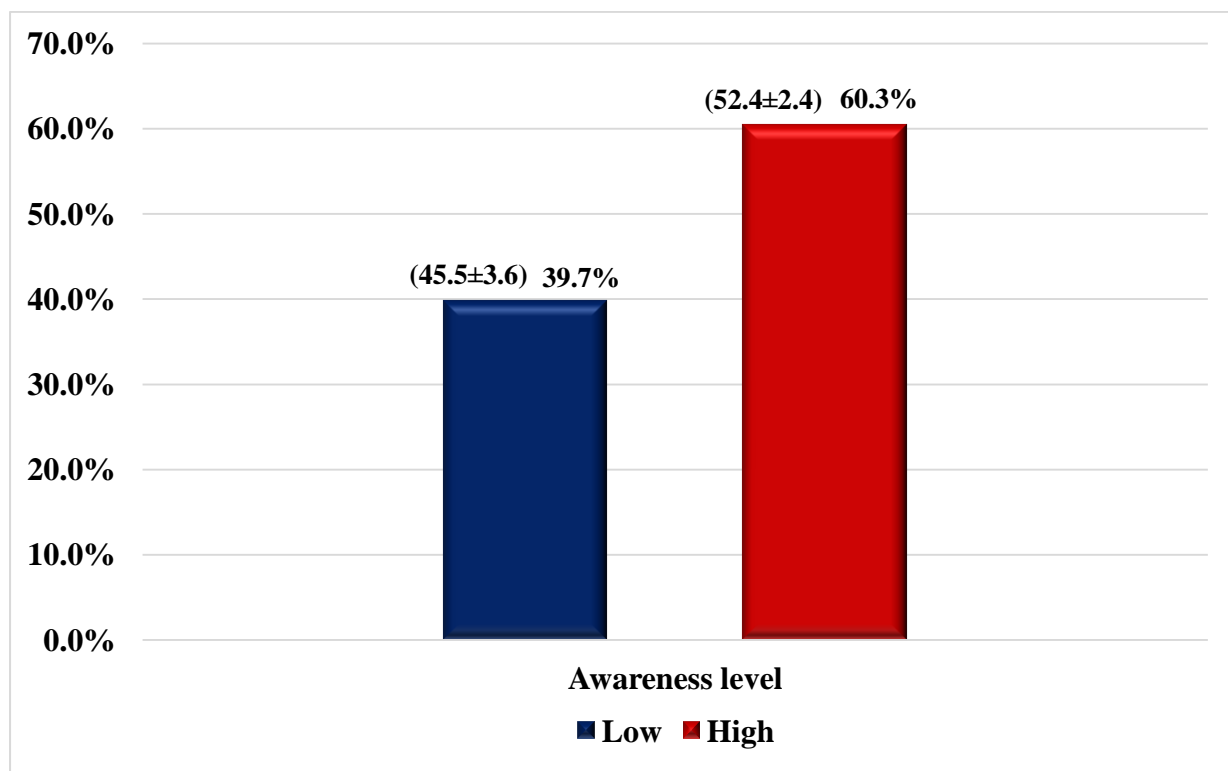

**Figure 1: Overall Level of Awareness of Laboratory Safety Measures**

Supplement: Supplementary file 4 — Additional file 4: Figure S1. Overall level of awareness of laboratory safety measures. [file 12879_2021_6806_MOESM4_ESM.pdf]

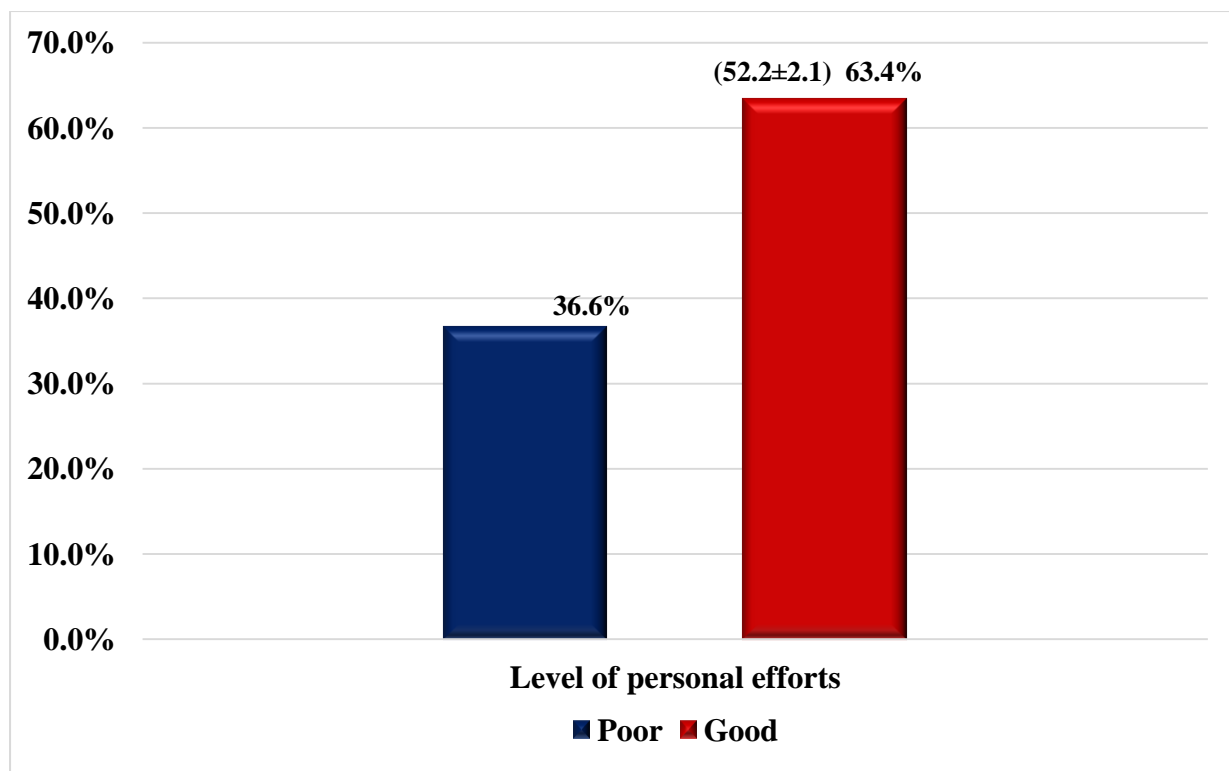

**Figure 2: Overall Level of Personal Efforts in Creating a Safe Laboratory Testing Environment.**

Supplement: Supplementary file 5 — Additional file 5: Figure S2. Overall level of personal efforts in creating a safe laboratory testing environment. [file 12879_2021_6806_MOESM5_ESM.pdf]
